# Metformin Use and Clinical Outcomes in Very Elderly Patients with Type 2 Diabetes and Chronic Kidney Disease

**DOI:** 10.3390/medicina62040776

**Published:** 2026-04-16

**Authors:** Michele Marchini

**Affiliations:** Azienda Sociosanitaria Ligure 5, 19124 La Spezia, Italy; michele.marchini@asl5.liguria.it

**Keywords:** chronic kidney disease, very elderly patients, metformin, type II diabetes, adverse clinical outcomes

## Abstract

*Background and Objactives:* Metformin is the most widely prescribed glucose-lowering therapy worldwide and is generally considered safe in patients with chronic kidney disease (CKD) with estimated glomerular filtration rate (eGFR) ≥30 mL/min/1.73 m^2^. However, very elderly patients are underrepresented in pivotal trials, and evidence on metformin safety in this vulnerable population remains limited. We evaluated the association between metformin use and adverse clinical outcomes in very elderly patients with CKD and type 2 diabetes. *Materials and Methods:* We conducted a single-center retrospective observational study including 624 very elderly patients (age > 78 years) with CKD, type 2 diabetes mellitus, and eGFR > 30 mL/min/1.73 m^2^. Patients were stratified according to metformin exposure (309 metformin-treated and 315 controls). The primary composite outcome was the first occurrence of intensive care unit (ICU) admission, initiation of renal replacement therapy (RRT), lactic acidosis, or all-cause mortality. A propensity score-matched sensitivity analysis and hierarchical win ratio analysis were also performed to further address potential baseline confounding. *Results:* Over a median follow-up of 33.7 months, the primary composite outcome occurred more frequently in the metformin group than in controls (18.7% vs. 9.5%; HR 1.75; 95% CI 1.12–2.73; *p* = 0.013). Metformin use was associated with a higher risk of ICU admission (HR 2.33; 95% CI 1.33–4.08), RRT initiation (HR 1.90; 95% CI 1.14–3.16), and lactic acidosis (HR 3.14; 95% CI 1.75–5.65). All-cause mortality was numerically higher but not statistically significant (HR 1.57; 95% CI 0.89–2.78). In a propensity score-matched analysis including 260 matched pairs, the association between metformin exposure and adverse outcomes remained consistent, and hierarchical win ratio analysis favored the control group (win ratio 2.00; 95% CI 1.24–3.47). *Conclusions:* In very elderly patients with CKD and type 2 diabetes, metformin use was associated with a higher observed risk of adverse clinical outcomes. These findings support a cautious, individualized risk–benefit assessment when prescribing metformin in this population.

## 1. Introduction

According to global estimates, approximately 700 million people worldwide are affected by chronic kidney disease (CKD). The prevalence of CKD has steadily increased since the 1990s, largely as a result of population growth and global population aging [1]. Despite the growing burden of chronic kidney disease in older adults, very elderly patients remain underrepresented in pivotal pharmacological trials. As a consequence, evidence on efficacy and safety derived from these trials is less robust in very elderly populations, who are frequently characterized by multimorbidity, frailty, and polypharmacy [2,3]. Metformin is the most widely prescribed antidiabetic drug worldwide; metformin exerts its glucose-lowering effect primarily by inhibiting hepatic gluconeogenesis, is characterized by a large volume of distribution due to minimal plasma protein binding, and is predominantly eliminated by renal excretion [4]. It is well known that as glomerular filtration rate (eGFR) declines below specific thresholds, metformin accumulation may occur, leading to an increased risk of lactic acidosis, namely metformin-associated lactic acidosis (MALA) and other important adverse events including the need for renal replacement therapy (RRT) and mortality [5,6,7]. Elderly patients, due to several and often concomitant risk factors such as increased susceptibility to dehydration, a higher incidence of acute clinical events, and exposure to polypharmacy that alters renal hemodynamics (i.e., renin–angiotensin–aldosterone system inhibitors (RAASi), are at increased risk of acute fluctuations in eGFR and acute kidney injury (AKI) [8,9,10]. Although regulatory recommendations contraindicate metformin use in patients with an eGFR <30 mL/min/1.73 m^2^ [11,12], no explicit contraindications have been established in the very elderly populations, and evidence supporting its safety remains limited [13,14]. The aim of this study was to evaluate the association between metformin use and adverse clinical outcomes in very elderly patients with CKD and type 2 diabetes.

## 2. Materials and Methods

### 2.1. Study Design and Population

We conducted a single-center, retrospective observational study involving very elderly patients (age > 78 years) with chronic kidney disease (CKD), type 2 diabetes mellitus, and an estimated glomerular filtration rate (eGFR) greater than 30 mL/min/1.73 m^2^. eGFR was calculated using the Berlin Initiative Study 1 (BIS1) equation, which has been specifically validated in older populations. Using electronic medical records, we identified eligible patients over a 10-year period (from 2015 to 2025). All consecutive patients meeting inclusion criteria during the study period were screened and included. A total of 624 patients were included and stratified according to metformin exposure (309 metformin-treated and 315 controls). The median follow-up duration was 33.7 months. Metformin exposure was defined based on baseline treatment status at study entry.

### 2.2. Inclusion and Exclusion Criteria

Inclusion criteria comprised very elderly patients (age > 78 years) with CKD, eGFR > 30 mL/min/1.73 m^2^, and type 2 diabetes mellitus. Exclusion criteria included kidney or any other solid organ transplantation and inflammatory or immune-mediated renal diseases, including systemic vasculitis, systemic lupus erythematosus, type 1 diabetes mellitus, IgA nephropathy, membranous nephropathy, and membranoproliferative glomerulonephritis. The study was conducted using fully anonymized routinely collected clinical data, in accordance with local institutional policy and the principles of the Declaration of Helsinki.

### 2.3. Outcome Definition

The primary composite outcome was the first occurrence of any of the following: intensive care unit (ICU) admission, defined as hospitalization requiring transfer to an ICU; renal replacement therapy (RRT), defined as the acute initiation of hemodialysis during follow-up; lactic acidosis was defined as arterial lactate levels >4 mmol/L, and all-cause mortality.

### 2.4. Statistical Analysis

Multivariable Cox proportional hazards regression was applied to evaluate the association between metformin exposure and outcomes, adjusting for clinically relevant covariates including age, sex, ischemic heart disease, peripheral artery disease, malignancy, and glycated hemoglobin (HbA1c) levels. Results were reported as hazard ratios (HRs) with 95% confidence intervals (CIs). A two-sided *p* value < 0.05 was considered statistically significant. Secondary outcomes consisted of the individual components of the composite endpoint. Secondary outcomes were considered exploratory and no adjustment for multiple comparisons was applied.

To further address potential baseline confounding, a propensity score-matched analysis was performed. Propensity scores for metformin exposure were estimated using a logistic regression model including age, sex, ischemic heart disease, peripheral artery disease, malignancy, and HbA1c levels. Patients treated with metformin were matched 1:1 with controls using nearest-neighbour matching without replacement and a caliper width of 0.2 of the standard deviation of the logit of the propensity score. Covariate balance after matching was assessed using standardized mean differences. In the matched cohort, a hierarchical win ratio analysis was performed prioritizing outcomes according to clinical severity in the following order: all-cause mortality, renal replacement therapy, ICU admission, and lactic acidosis. Ninety-five percent confidence intervals were estimated using bootstrap resampling. Missing data were minimal and handled using a complete-case analysis.

Statistical analyses were performed using MedCalc Statistical Software version 23.4.5 (MedCalc Software Ltd., Ostend, Belgium).

## 3. Results

### 3.1. Baseline Characteristics

Baseline characteristics of the study population are reported in Table 1. Metformin exposure was defined based on baseline treatment status. When available, data on concomitant pharmacological therapies were also collected.

### 3.2. Primary Outcome

**Figure 1:**
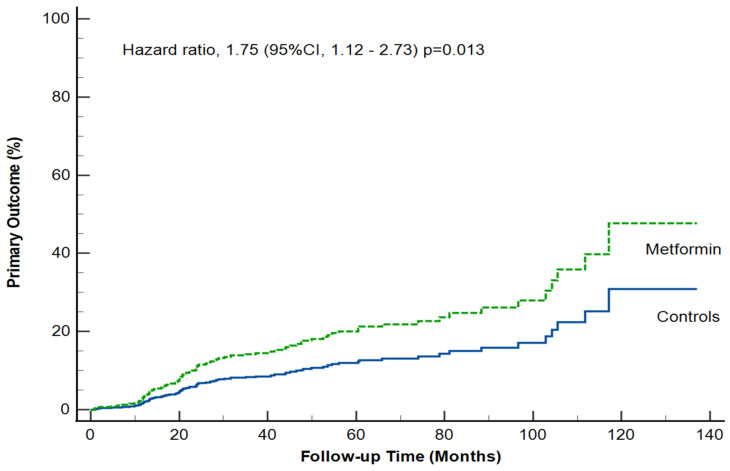
**Primary composite outcome.** Estimated cumulative incidence (%) of the primary outcome with the use of Cox proportional-hazards regression model, adjusted for age, sex, ischemic heart disease, peripheral artery disease, malignancy, HbA1c (%) levels. The primary outcome was a composite of ICU admission, renal replacement therapy, lactic acidosis, and death for any cause.

The primary composite outcome (ICU admission, RRT initiation, lactic acidosis, or all-cause mortality) occurred in 58 of 309 patients (18.7%) in the metformin group and in 30 of 315 patients (9.5%) in the control group, HR 1.75; (95% CI 1.12–2.73); *p* = 0.013, corresponding to 5.15 and 2.92 events per 100 patient-years, respectively (Table 2 and Figure 1).

### 3.3. Secondary Outcomes

**Figure 2:**
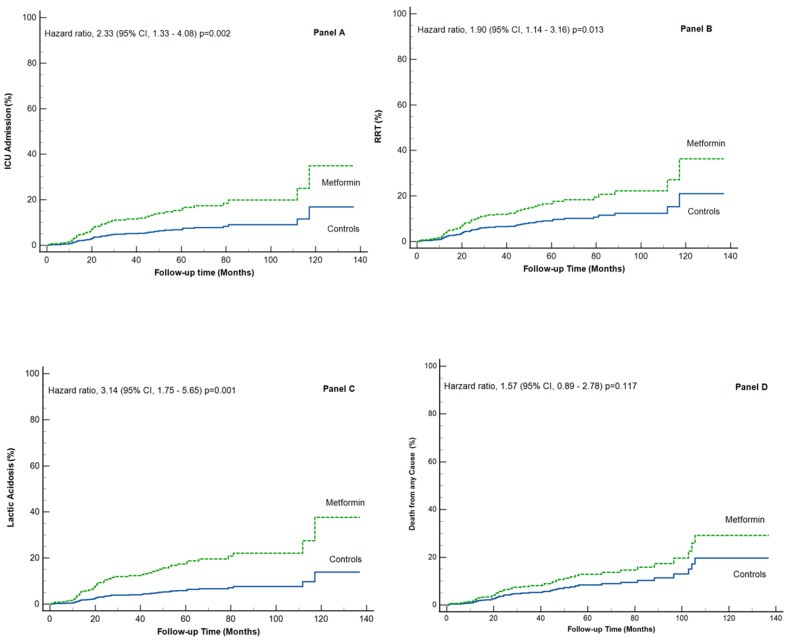
**Secondary outcomes.** Estimated cumulative incidence of secondary outcomes with the use of Cox proportional-hazards regression model, adjusted for age, sex, ischemic heart disease, peripheral artery disease, malignancy, HbA1c (%) levels. Panel (**A**). ICU admission. Panel (**B**). Renal replacement therapy (RRT). Panel (**C**). Lactic acidosis. Panel (**D**). Death from any cause.

Similar patterns were observed across individual components of the composite endpoint. ICU admission occurred in 43 of 309 patients (13.9%) in the metformin group and in 18 of 315 patients (5.7%) in controls, HR 2.33; (95% CI 1.33–4.08); *p* = 0.002, corresponding to 4.0 and 1.7 events per 100 patient-years, respectively (Table 2 and Figure 2A).

The need for acute RRT was observed in 46 of 309 patients (14.8%) compared with 23 of 315 patients (7.3%), HR 1.90; (95% CI 1.14–3.16); *p* = 0.013, corresponding to 4.1 and 2.3 events per 100 patient-years, respectively (Table 2 and Figure 2B).

Lactic acidosis developed in 48 of 309 patients (15.5%) in the metformin group versus 15 of 315 patients (4.7%) in controls, HR 3.14; (95% CI 1.75–5.65); *p* = 0.001, corresponding to 4.3 and 1.5 events per 100 patient-years, respectively (Table 2 and Figure 2C).

All-cause mortality was numerically higher in the metformin group but did not reach statistical significance, 33 of 309 (10.6%) vs. 19 of 315 (6.0%); HR 1.57; (95% CI 0.89–2.78); *p* = 0.117 (Table 2 and Figure 2D).

### 3.4. Propensity Score-Matched Analysis

To further support these findings, a propensity score-matched sensitivity analysis was performed, identifying 260 matched pairs of patients receiving metformin and controls. Baseline characteristics were well balanced after matching across all variables included in the propensity score model.

In the matched cohort, the primary composite outcome occurred in 48 of 260 patients (18.5%) in the metformin group and in 27 of 260 patients (10.4%) in the control group.

In a hierarchical analysis prioritizing mortality, renal replacement therapy, ICU admission, and lactic acidosis, win ratio analysis favored the control group, with 46 wins versus 23 wins in the metformin group (191 ties), corresponding to a win ratio of 2.00 (95% CI 1.24–3.47).

## 4. Discussion

We found that very elderly patients with chronic kidney disease with type 2 diabetes who receive metformin had a higher risk of the primary composite outcome of admission to ICU, need for renal replacement therapy (RRT), lactic acidosis and all-cause mortality compared to elderly patients who did not take metformin. Each of the components, except for all-cause mortality, of the composite outcome occurred more frequently in the metformin group. These findings suggest that, in very elderly patients with moderate CKD, the risk profile of metformin may differ substantially from that reported in younger or less comorbid populations traditionally enrolled in randomized trials. Importantly, the association remained consistent in a propensity score-matched sensitivity analysis, suggesting that the observed relationship between metformin exposure and adverse outcomes is unlikely to be explained solely by baseline differences between treatment groups. In our study, all patients were older than 78 years, with a mean age of 81.2 years (range 78–96), representing a population in whom evidence regarding a favorable benefit–risk profile remains limited. Clinical and pathophysiological evidence suggests that older adults are more vulnerable to drug-related adverse events, due to age-related changes in pharmacokinetics and pharmacodynamics, multimorbidity, and polypharmacy [15,16]. This vulnerability is further amplified in patients with chronic kidney disease, in whom reduced drug clearance and fluctuating renal function due to intercurrent clinical events predispose to drug accumulation and toxicity [17,18]. The most clinically relevant adverse event associated with metformin therapy is metformin-associated lactic acidosis (MALA), which frequently requires hospitalization and is commonly accompanied by acute kidney injury or acute-on-chronic kidney dysfunction, often necessitating renal replacement therapy—particularly continuous renal replacement therapy (CRRT)—and is associated with high mortality rates [19,20,21]. Notably, the excess risk observed in metformin-treated patients was mainly driven by outcomes that are mechanistically consistent with metformin accumulation and acute renal function deterioration, namely lactic acidosis, ICU admission, and the need for renal replacement therapy, rather than by a nonspecific increase in overall mortality alone. This occurs because metformin, which is predominantly eliminated by the kidneys and characterized by a large volume of distribution due to minimal plasma protein binding, tends to accumulate during acute or acute-on-chronic deterioration of renal function [22]. Studies actually showed how the risk of MALA gets higher when kidney function falls below a certain threshold [23,24]. Nevertheless, large observational studies and systematic reviews have reported that the absolute risk of lactic acidosis is low in the general population with a mild reduction or normal renal function and emphasized the substantial clinical benefits of metformin therapy [25,26,27].

However, these studies have rarely focused on particularly vulnerable populations such as the very elderly, in whom the balance between risks and benefits may substantially differ.

This apparent discrepancy with previous studies may reflect the selection of healthier and younger individuals in randomized trials and large observational cohorts, limiting the generalizability of those findings to frail very elderly patients with CKD, who more frequently experience acute changes in renal function. While metformin has consistently demonstrated cardiovascular and survival benefits in younger and less comorbid populations, our findings suggest the need for heightened caution when prescribing metformin in very elderly patients with CKD, even when eGFR remains above conventional safety thresholds. Our findings should not be interpreted as evidence against metformin use in general, but rather as an indication that its safety profile may differ in very elderly patients with chronic kidney disease, a population largely underrepresented in clinical trials. Closer monitoring of renal function, prompt drug discontinuation during acute illness, and individualized risk–benefit, beyond the only reliance on renal function thresholds, may be particularly warranted in this high-risk population. Our study presents several limitations that are inherent to its retrospective observational design. The lack of randomization and the possibility of residual confounding preclude causal inference, and these findings can only be regarded as hypothesis generating. Despite multivariable adjustments and propensity score matching, residual confounding by indication cannot be excluded, particularly in a very elderly population where factors such as frailty, functional status, and acute intercurrent illnesses may influence both treatment allocation and outcomes. The only reliance on electronic medical records may have led to misclassification of exposures or outcomes, and some clinical variables were not consistently available for all patients during their observation time. Unmeasured confounders such as severity of comorbidities, and medication adherence could not be fully accounted for. As this was a retrospective observational study, no formal a priori sample size calculation was performed. Nevertheless, the number of observed events was sufficient to support multivariable time-to-event analyses, with an events-per-variable ratio of approximately 12.6 (88 events across seven model parameters), exceeding commonly recommended thresholds for multivariable Cox modeling. Despite these limitations, the study demonstrated a statistically significant association with an effect size likely to be clinically relevant in this high-risk population, supported by a plausible biological and pharmacological rationale.

## 5. Conclusions

As the global population is progressively aging, evaluating the effectiveness and safety of therapeutic interventions in older adults, who are frequently underrepresented in clinical trials, has become increasingly important. In this context, greater effort should be devoted to discerning when a treatment is truly clinically beneficial and when it may instead be harmful, as well as to weighing potential benefits against the risk of adverse events. Future prospective studies specifically designed to evaluate drug safety in very elderly patients with CKD are needed to better define when the potential benefits of metformin outweigh the risks in this particularly vulnerable population. Only in this way can we ensure adherence to one of the fundamental principles of medicine, “primum non nocere”.

## Figures and Tables

**Table 1:** Demographic and clinical characteristics of the participants at baseline.

Characteristic	Metformin (N = 309)	Controls (N = 315)
Age *	80.8 ± 4.0	81.7 ± 4.0
Female sex—no. (%)	132 (42.7)	122 (38.7)
Male sex—no. (%)	177 (57.3)	191 (60.6)
eGFR ‡		
Mean—ml/min/1.73 m^2^	37.7 ± 7.7	37.2 ± 7.6
Distribution—no. (%)		
≥60 mL/min/1.73 m^2^	12 (4.0)	12 (3.8)
45 to <60 mL/min/1.73 m^2^	17 (5.5)	15 (4.7)
30 to <45 mL/min/1.73 m^2^	285 (90.5)	290 (91.5)
Type II Diabetes Mellitus—no. (%)	309 (100)	315 (100)
HbA1c Mean—(%)	7.2%	7.4%
Hemoglobin *—gr/L	116.7 ± 15.1	113.6 ± 18.1
Urinary albumin-to-creatinine ratio—no. (%)		
<1000 mg/gr	246 (79.6)	258 (81.9)
>1000 mg/gr	63 (20.4)	57 (18.1)
Ischemic Heart Disease ¶—no. (%)	33 (10.6)	36 (11.4)
Peripheral Artery Disease §—no. (%)	16 (5.2)	12 (3.8)
Malignancy †—no. (%)	34 (11.0)	19 (6.0)
Previous medication—no. (%)		
ACE inhibitor	123 (39.8)	131 (41.5)
ARB	103 (33.3)	117 (37.1)
SGLT2i	19 (6.0)	16 (5.0)
Diuretic	98 (31.7)	114 (36.1)
Statin	108 (34.9)	121 (38.4)
Metformin dose—no. (%)		
<1000 mg	78 (25.4)	N/A
>1000; <2000 mg	198 (64.0)	N/A
>2000 mg	33 (10.6)	N/A

* Plus–minus values are mean ± SD. ¶ Ischemic heart disease was defined as a history of, myocardial infarction, percutaneous coronary intervention, coronary-artery bypass grafting. § Peripheral artery disease was defined as a history of lower limb percutaneous luminal angioplasty or stenting or surgical amputation for vascular reasons. † Malignancy was defined as biochemical, histological or radiological evidence of malignancy. ‡ eGFR was estimated using BIS1 equation. ACE denotes angiotensin-converting enzyme, ARB angiotensin-receptor blocker, and eGFR estimated glomerular filtration rate.

**Table 2:** Primary and secondary outcomes.

Outcome	Metformin *no./Total no.(%) Events/100* *Patient-yr*		Controls *no./Total no.(%) Events/100* *Patient-yr*		Hazard Ratio (95%CI)	*p* Value *
Primary Outcome	58/309 (18.7)	5.15	30/315 (9.5)	2.92	1.75 (1.12–2.73)	0.013
Secondary Outcomes						
ICU Admission	43/309 (13.9)	4.0	18/315 (5.7)	1.7	2.33 (1.33–4.08)	0.002
RRT	46/309 (14.8)	4.1	23/315 (7.3)	2.3	1.90 (1.14–3.16)	0.013
Lactic Acidosis	48/309 (15.5)	4.3	15/315 (4.7)	1.5	3.14 (1.75–5.65)	0.001
Death From Any Cause	33/309 (10.6)	2.9	19/315 (6.0)	1.9	1.57 (0.89–2.78)	NS

* *p* value is shown if <0.05.

## Data Availability

The data supporting the findings of this study contain identifiable clinical information and are therefore not publicly available. Anonymized data may be made available by the corresponding author upon reasonable request and subject to institutional regulations.
